# Stem cell transplantation in stroke: Current obstacles and future directions to improve efficacy

**DOI:** 10.4103/NRR.NRR-D-25-01155

**Published:** 2026-01-27

**Authors:** Abdullah Md. Sheikh, Shozo Yano, Atsushi Nagai

**Affiliations:** Department of Laboratory Medicine, Faculty of Medicine, Shimane University, Izumo, Japan; Department of Neurology, Faculty of Medicine, Shimane University, Izumo, Japan

Cerebral stroke, particularly the ischemic type, is one of the leading causes of disability and mortality worldwide. Alarmingly, the incidence of ischemic stroke is rising, especially among younger populations (Zhang et al., 2025). This concerning trend highlights the urgent need to develop and improve therapeutic strategies for stroke prevention and management. Current disease-modifying treatments for ischemic stroke primarily focus on restoring blood flow by dissolving the obstructing clot using enzymes such as tissue plasminogen activator or through mechanical thrombectomy. However, these interventions are limited by a narrow therapeutic time window, rendering a large proportion of patients ineligible. As a result, many stroke survivors experience long-term neurological deficits. These patients could benefit from regenerative therapies aimed at restoring damaged neural tissue. Even partial recovery could improve the quality of life of the patient group, also reduce the burden on health system. In this context, we propose a comprehensive regenerative approach for stroke using a combination therapy that includes sequential transplantation of genetically engineered, multiple cell types, along with gene therapy to promote neural differentiation. We also addressed the potential causes of poor grafting efficiency of transplanted cells and discussed strategies to overcome these challenges. This therapeutic strategy can be evaluated using animal models of stroke. We believe that such an approach could provide a more effective regenerative treatment option for stroke patients.

**Figure 1:**
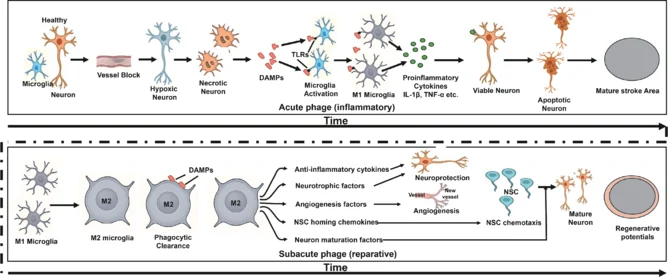
Time-dependent pathological changes in stroke. Following ischemia caused by the blockage of blood vessels, neural tissues experience hypoxia and ultimately undergo necrotic cell death. This event triggers an acute inflammatory phase. The necrotic tissues release DAMPs, which activate microglia through pattern recognition receptors such as TLRs, promoting their polarization to the pro-inflammatory M1 phenotype. These activated M1 microglia release a range of pro-inflammatory cytokines, chemokines, and enzymes, including IL-1β, TNF- α, MCP-1, and iNOS, which recruit additional inflammatory cells, stimulate their activation, and promote the production of reactive oxygen species. This inflammatory environment exacerbates neuronal death, particularly through apoptosis in the ischemic border zones where perfusion is compromised. In the subsequent subacute reparative phase, microglia transform to M2 type, which is associated with tissue repair. M2-type microglia are phagocytic and contribute to the clearance of dead cells and debris, thereby reducing inflammatory signaling. Additionally, they secrete anti-inflammatory cytokines and neurotrophic factors that support neuronal survival. They also produce chemokines that attract NSCs and secrete factors that promote their maturation into functional neurons. Furthermore, they release angiogenic factors that stimulate the formation of new blood vessels within the lesion area, enhancing perfusion and supporting both injured tissues and newly generated neurons. The components of the figures are generated using DALL-E, and then assembled using Microsoft PowerPoint. DAMPs: Damage-associated molecular patterns; IL: interleukin; iNOS: inducible nitric oxide synthase; MCP-1: monocyte chemoattractant protein-1; NSCs: neural stem cells; TLRs: Toll-like receptors; TNF: tumor necrosis factor.

Ischemic stroke typically results from the blockage of a cerebral artery, most commonly due to a thrombus or embolus. Such blockage immediately leads to reduced cerebral perfusion, energy failure, disruption of ion homeostasis, and subsequent cytotoxic edema. The disrupted ion homeostasis also promotes glutamate-mediated excitotoxicity, which is a major mechanism of early neuronal death. In addition, ischemia leads to the overproduction of reactive oxygen species-mediated tissue damage. Finally, necrotic death occurs in the brain tissue supplied by the affected artery. The necrotic tissue and associated insults, in turn, trigger a robust inflammatory response, mediated by damage-associated molecular patterns (DAMPs) and the activation of resident microglia and infiltrating immune cells (Haupt et al., 2024). These inflammatory signals, and oxidative stress combined with compromised blood supply in the surrounding ischemic penumbra contributes to secondary injury, leading to apoptotic death of neurons and glial cells, causing expansion of the lesion area over time. In the subacute to chronic stages, astrocytes become activated and form a glial scar, effectively walling off the lesion from surrounding healthy tissue. The time-dependent changes in the molecular pathology of ischemic stroke are shown in **Figure 1**.

In addition to destructive processes, several endogenous reparative mechanisms are activated following stroke in an attempt to limit tissue damage and promote recovery (Ruan et al., 2015; Marques et al., 2019). One such mechanism is the induction of angiogenesis in response to hypoxia. The hypoxic environment within the ischemic core and penumbra stimulates the expression of angiogenic factors, such as vascular endothelial growth factor (Ruan et al., 2015), leading to the formation of new blood vessels for the restoration of perfusion. However, the newly formed vasculature often lacks structural and functional integrity, contributing to increased vascular permeability and cerebral edema. Another critical reparative response involves phenotypic changes in microglia. During the acute phase of stroke, microglia predominantly adopt a proinflammatory M1 phenotype, characterized by the release of cytokines such as tumor necrosis factor α and interleukin (IL)-1β, which can exacerbate neuronal injury (Haupt et al., 2024). Some of the microglia undergo a shift toward the M2 phenotype, which is associated with anti-inflammatory, neuroprotective, and phagocytic functions. For such functions and repair of the damaged brain tissue after stroke, M2 microglia produce several anti-inflammatory cytokines, angiogenic, neurotrophic, and neuroprotective factors. Importantly, phagocytic functions play a key role in modulating the inflammatory response through the clearance of cellular debris. Additionally, the stroke-injured brain exhibits activation and migration of endogenous neural stem cells (NSCs) from neurogenic niches, such as the subventricular zone, toward the lesion site. This migration is thought to be influenced by various signals in the injury microenvironment, including chemokines, growth factors, and possibly M2-type microglia. Evidence suggests that microglia may secrete NSC-directed chemoattractants such as CXCL12, which also support the survival and differentiation of NSC, thereby contributing to the repair of the damaged neural tissues.

Despite these endogenous efforts, the regenerative capacity of the adult brain remains limited. Neither angiogenesis, microglia-mediated neuroprotection, nor NSC-driven neurogenesis is sufficient on its own to fully, or at least substantially, repair the infarcted area and restore lost neurological function. Nonetheless, understanding these mechanisms provides a foundation for the development of therapeutic strategies that can target and enhance the natural repair pathways. Such potential strategies include: (a) Enhancement of angiogenesis, (b) Modulation of neurotrophic and growth factors, (c) Chemokine modulation to increase NSC recruitment, (d) Stem cell-based therapies, (e) Microglia-based therapy and modulation of microglial phenotypes, and (f) Microenvironmental modulation. Our previous studies showed that mesenchymal stem cell transplantation improves ischemic stroke pathology by enhancing angiogenesis and neurotrophic support while reducing inflammation (Sheikh et al., 2011, 2019). Mesenchymal stem cells also have the ability to transform microglia to anti-inflammatory, neuroprotective, and angiogenic M2 type (Liu et al., 2019). Along with the modulation of neuroinflammation, angiogenesis, and neurotropic support, mesenchymal stem cells also promote early NSC migration into the lesion. However, the mobilization of endogenous NSCs remains insufficient for fully replacing damaged neurons or restoring functional neural circuits. Given that strategies, such as promoting angiogenesis, dampening inflammation, and supporting endogenous NSC migration, yield only partial recovery, exogenous NSC transplantation may offer an alternative for tissue replacement. Transplanted NSCs have been shown to accumulate in the stroke-affected areas, and also have the ability to decrease local inflammatory conditions. Although their accumulation results in some improvement, the success has been limited by poor homing of exogenous NSC, as well as their limited survival and integration with host tissue. Hence, the observed improvements in stroke pathology by the NSC transplantation may stem more from modulation of the inflammatory microenvironment than from actual graft incorporation.

A major barrier to successful NSC grafting is the hostile microenvironment at the lesion site, marked by elevated levels of proinflammatory cytokines and DAMPs, which severely compromise NSC survival. Inadequate angiogenesis and insufficient trophic support further contribute to the widespread death of transplanted cells. Impaired differentiation into mature neurons also plays a role, as undifferentiated or improperly differentiated NSCs are prone to apoptosis. Successful integration of NSCs requires local cues to direct differentiation, axonal growth, synapse formation, and the establishment of functional neural circuits. After maturation, the absence of supportive structural and chemical signals, such as extracellular matrix components and guidance molecules, hinders neurons both integration and circuit formation, ultimately causes apoptotic death. To overcome such obstacles, one promising strategy would be prior to transplantation of NSC, the induction of the transformation of microglia to M2-type. M2-type microglia possess anti-inflammatory and neuroprotective properties, resulting in a reduction of DAMP- and cytokine-induced inflammation. Such reduction of DAMPs and proinflammatory cytokines could create a favorable microenvironment for survival of exogenous and endogenous NSCs, and integration to the host tissue. However, *in vivo* transformation of the microglia to the M2 phenotype remains challenging. The transcription factor STAT6 plays a central role in M2 polarization and can be activated by cytokines, such as IL-4 and IL-13. Additionally, PPARγ agonists such as rosiglitazone have been shown to promote *in vivo* M2 polarization and reduce infarct size in stroke models (Han et al., 2015). Nevertheless, systemic administration of such cytokines or drugs may lead to unintended side effects. To overcome these limitations, we propose an alternative strategy: transforming microglia into the M2 phenotype *in vitro*, followed by transplantation into the stroke-affected brain, and then transplantation of NSCs. This approach offers greater control over microglia M2 transformation efficiency and reduces systemic exposure to modulatory agents. Once transplanted, M2 microglia can phagocytose DAMPs, reduce proinflammatory signaling, and secrete anti-inflammatory cytokines such as IL-4. Importantly, this secreted IL-4 can promote further M2 polarization of endogenous microglia and amplify the therapeutic effects. Additionally, M2 microglia release angiogenic molecules such as vascular endothelial growth factor, basic fibroblast growth factor, and chemokines such as CXCL2 that support endothelial cell migration (Haupt et al., 2024). They also secrete factors such as Ym1/2, IL-10, and transforming growth factor β, which can enhance blood-brain barrier integrity during angiogenesis process and support the formation of functional vessels. In stroke, however, newly formed vessels often lack proper blood-brain barrier and other structural supports, making them the cause of vasogenic edema that worsens the situation. Hence, the formation of functional vessels supported by M2 microglia secreted factors not only improves the vascular stability and perfusion, but also alleviates the edema condition, which is frequently seen in stroke. These pro-angiogenic effects help improve the lesion microenvironment, and subsequent transplantation of NSCs would have a better chance of survival.

M2 microglia has been shown to stimulate endogenous NSC proliferation and neuronal differentiation, which would confer an additive benefit to the transplanted NSCs (Song et al., 2023). However, the glial scar can hinder the NSC-mediated regenerative process by making physical barriers as well as secreting growth-inhibitory molecules such as chondroitin sulfate proteoglycans. A previous study demonstrated that M2 microglia, particularly via their extracellular vesicles, can modulate glial scar formation (Li et al., 2021). Another microenvironmental change necessary is an appropriate extracellular matrix composition that directs the differentiation of NSCs to relevant types of mature neurons, proper axonal projection, as well as supports the survival of the neurons and associated glial cells. While they do not directly produce most extracellular matrix proteins, M2 microglia influence astrocytes to synthesize extracellular matrix components that support neuronal repair and structural remodeling. Hence, preconditioning the lesion environment with M2-type microglia can address many of the key barriers to subsequent NSC grafting, particularly by controlling inflammation, enhancing angiogenesis, and providing neurotrophic support. These actions contribute to the establishment of a more favorable niche for NSC homing, survival, differentiation, and eventual integration into functional neural circuits.

Although M2-type microglia can improve the survival of NSCs in stroke-affected areas and possess some capacity to influence NSC differentiation into neurons, their ability alone may not be sufficient to drive full neuronal maturation and functional integration into existing neural circuits. Neuronal maturation is a highly complex and finely regulated process, with different neuronal subtypes requiring distinct extrinsic and intrinsic cues for proper development and circuit incorporation. Given that neural tissue is composed of a heterogeneous population of neurons, precise differentiation and connection guidance during maturation is essential to ensure appropriate structural and functional outcomes. Most of our current understanding of neuronal maturation has been derived from *in vitro* studies, where researchers employ various transcription factors, such as neurogenin 2, Ascl1, Brn2, Myt1l, and NeuroD1, to induce and enhance neuronal differentiation and maturation (Wapinski et al., 2013). Additionally, signaling pathways such as Sonic hedgehog, fibroblast growth factor (FGF)8, FGF17, FGF18, and Wnt1 are commonly used to mimic developmental conditions and promote specific neuronal fates. The selection of these transcription factors and signaling molecules stems from the insightful knowledge of developmental neurobiology, where distinct brain regions express unique combinations of these regulatory proteins. These signaling molecules often form spatiotemporal concentration gradients, which are crucial for guiding neuronal subtype specification and regional identity during development. Based on these principles, numerous protocols have been developed for *in vitro* NSC differentiation. These models are used not only to study disease mechanisms but also to establish drug targets and test the efficacy of candidate therapeutics. Not only the *in vitro* cell culture systems, recent studies also demonstrate that transcription factor modulation can be used for neuronal reprogramming *in vivo* in animal disease models. For example, NeuroD1-mediated gene therapy has been shown to induce conversion of reactive astrocytes into neurons following brain injury (Puls et al., 2020). Studies also showed that chemical reprogramming of astrocytes to neurons can be done *in vitro* cell culture system using the combination of small molecules that are applied to the culture at specific differentiation stages. Such small molecule cocktails usually facilitate the transdifferentiation through the regulation of transcription factors such as NeuroD1 (Zhang et al., 2015). However, achieving astrocyte-to-neuron reprogramming using small molecules *in vivo* might be challenging. Small molecules used for transdifferentiation may have some off-target effects, and their optimal concentrations and delivery systems need to be carefully tailored to maximize efficiency while minimizing adverse outcomes. Moreover, methods to track neuronal differentiation stages *in vivo* are required to be developed for the temporal selection of appropriate small molecules according to the developmental stages. In addition, astrocyte-to-neuron transdifferentiation mediated by small molecules generally occurs at lower efficiency than NeuroD1 gene therapy. Studies using adeno-associated virus–based NeuroD1 delivery systems have reported neuronal conversion rates of up to 90% of reactive astrocytes expressing the ectopic gene, whereas the efficiency of small molecules was around 65%. Nevertheless, small molecule-based transdifferentiation of astrocytes into neurons could provide a promising alternative to gene therapy-based approaches.

**Figure 2:**
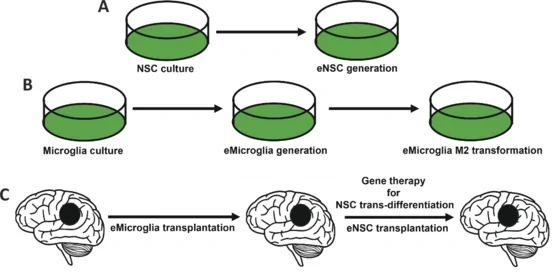
Transplantation-based stroke therapy strategy. This transplantation-based stroke therapy involves a two-step approach: first, preparing the stroke-affected areas for NSC grafting, and second, transplanting the NSCs. The NSCs would be genetically engineered to differentiate into specific types of neurons (A), tailored to the neuronal composition of the target brain region. Because each brain area contains unique neuronal subtypes, transplantation of lineage-specific NSCs can help restore the structural and functional integrity of the affected area. To support the differentiation of these specific neuronal types, microglia would also be genetically modified to produce defined neuronal differentiation modulators (B). These engineered microglia would be polarized to the reparative M2 phenotype *in vitro* prior to transplantation. (C) In the acute phase post-stroke, M2-type microglia would be transplanted first to clear necrotic debris through phagocytosis, reduce inflammation, and release neurotrophic factors, thereby creating a favorable microenvironment for subsequent NSC engraftment. Transplantation of the genetically modified NSCs into this primed environment would enhance their survival, integration, and differentiation. Moreover, chemokines secreted by the M2 microglia would recruit endogenous NSCs, further enhancing the regenerative process. Angiogenic factors released by these microglia would promote neovascularization, improving perfusion and nutrient supply to support the survival and function of newly integrated cells. Importantly, the neuron differentiation modulators produced by the genetically modified microglia would help guide both transplanted and endogenous cells, including astrocytes, toward appropriate neuronal and glial fates. In parallel with cell transplantation therapy, gene therapy could be employed to induce trans-differentiation of resident astrocytes into neurons. This comprehensive therapeutic approach aims to enhance the regenerative capacity of the brain and significantly improve functional recovery after stroke. The components of the figures are generated using DALL-E, and then assembled using Microsoft PowerPoint. eMicroglia: Engineered microglia; eNSC: engineered neural stem cell; NSC: neural stem cell.

Several transcription factors, including NeuroD1, play important roles in functional neuron reprograming (Puls et al., 2020). NeuroD1 is particularly important for the differentiation and maturation of neural progenitor cells into cortical pyramidal neurons and granule cells in the hippocampus and cerebellum, highlighting its role in shaping region-specific neuronal identities. Several other transcription factors are also implicated in neuronal maturation. For example, the induction of transcription factors such as NGN2 or ASCL1/DLX2, in combination with diverse signaling molecules, can differentiate pluripotent stem cell–derived neurons into various neuronal subtypes (Lin et al., 2025). *In vivo* and *in vitro* expression of NGN2 in oligodendrocyte progenitor cells was found to generate mature cortical pyramidal neurons with spiny dendrites and axons, forming proper synaptic connections, and exhibiting appropriate subtype specifications aligned with the local cytoarchitecture (Bazarek et al., 2023). Similarly, ASCL1 has been implicated in the maturation of hippocampal granule cells. In this context, NSCs can be reprogrammed or engineered (engineered NSC [eNSC]) *in vitro* to create cell lines stably expressing these transcription factors, and depending on the region-specific neuron types that are required to be replaced, the NSC line can be chosen for transplantation (**Figure 2A**). Depending on the desired mature neuron type, signaling molecules can be selected that work in combination with transcription factors expressed in the transplanted NSCs, thereby directing their differentiation into specific neuronal subtypes required for regeneration. The delivery system of signaling molecules also needs to be perfected for proper and optimized delivery at a proper time that is necessary for neuronal differentiation. Such tailored approaches are crucial for achieving accurate neuronal replacement and functional recovery.

While neuronal reprogramming is a promising strategy, it must be complemented by strategies that support the generation of astrocytes to ensure the formation of a balanced and supportive neural microenvironment. Astrocyte differentiation also relies on key transcriptional regulators. NFIA and ATF3 have been identified as critical for astrocyte specification from neural progenitors, while RUNX2 promotes astrocyte maturation (Tiwari et al. 2018). Given the essential roles of both neurons and astrocytes in maintaining brain homeostasis and function, therapeutic strategies should aim to promote the proper differentiation and maturation of both cell types. For the generation of an astrocyte cell population, stem cell-based therapy may be a more suitable approach, as viral vector-mediated gene therapy could interfere with simultaneous neuronal differentiation. Hence, strategies such as generating stem cell lines reprogrammed to express astrocyte-specific transcription factors and transplanting them alongside neuron-directed NSCs may be an effective approach for achieving proper regeneration.

Based on these findings, we propose a multimodal therapeutic strategy for stroke that combines: (1) M2-type microglia transplantation, which not only clears the DAMPs, makes a suitable environment for NSC grafting, provides trophic support and anti-inflammatory effects, but also secretes beneficial signaling molecules; (2) NSC or neural progenitor cell transplantation, with careful selection or engineering to bias differentiation toward desired neuronal or glial fates. Gene therapy approaches using transcription factors, such as NeuroD1 would be helpful to promote endogenous and transplanted neuronal maturation; (3) delivery of signaling molecules that stimulate and recapitulate the developmental milieu of astrocytes and neuronal differentiation and maturation.

Moreover, M2-type microglia can be genetically modified engineered (engineered microglia [eMicroglia]) to enhance or direct specific signaling pathways, further optimizing the local environment for neural repair (**Figure 2B** and **C**). This integrated strategy, combining cellular, genetic, and molecular interventions, holds promise for improving the efficacy of stroke therapy by promoting the regeneration of a functionally diverse and properly organized neural network.

In conclusion, a comprehensive therapeutic strategy that combines M2-type microglia transplantation, NSC-based replacement, targeted gene therapy, and signaling molecule delivery may offer a promising approach for stroke recovery. By modulating the lesion microenvironment, enhancing neuroprotection, and guiding cell fate decisions, this multimodal approach can improve NSC survival, differentiation, and integration. Tailoring these interventions to regional neuronal identities and incorporating supportive glial components is necessary for rebuilding functional neural circuits and achieving a significant neurological restoration following stroke.
